# Diagnostic Value of an Additional Sequence (Large-Field Coronal Stir) in a Routine Lumbar Spine MR Imaging Protocol to Investigate Lumbar Radiculopathy

**DOI:** 10.3390/jcm12196250

**Published:** 2023-09-28

**Authors:** Quentin Patriat, François-Victor Prigent, Serge Aho, Marc Lenfant, André Ramon, Romaric Loffroy, Aurelien Lambert, Paul Ornetti

**Affiliations:** 1 Department of Vascular and Interventional Radiology, François-Mitterrand University Hospital, 21079 Dijon, France; quentin.patriat@gmail.com (Q.P.); francois-victor.prigent@chu-dijon.fr (F.-V.P.); romaric.loffroy@chu-dijon.fr (R.L.); 2 Department of Epidemiology and Biostatistics, François-Mitterrand University Hospital, 21079 Dijon, France; serge.aho@chu-dijon.fr; 3 Department of Neuroradiology and Emergency Radiology, François-Mitterrand University Hospital, 21079 Dijon, France; marc.lenfant@chu-dijon.fr; 4 Department of Rheumatology, François-Mitterrand University Hospital, 21079 Dijon, France; andre.ramon@chu-dijon.fr; 5 INSERM, EFS Bourgogne Franche-Comté, UMR 1098, RIGHT Graft-Host-Tumor Interactions/Cellular and Genetic Engineering, Bourgogne Franche-Comté University, 21079 Dijon, France; 6 ICMUB Laboratory, UMR CNRS 6302, University of Burgundy, 9 Avenue Alain Savary, 21079 Dijon, France; 7 Department of Radiology, IM2P, Clinique Valmy, 21079 Dijon, France; aurelien.lambert@im2p.fr; 8 INSERM UMR1093-CAPS, Bourgogne Franche-Comté University, UFR STAPS, 21079 Dijon, France; 9 INSERM, Bourgogne Franche-Comté University, CIC 1432, Module Plurithématique, Plateforme d’Investigation Technologique, François-Mitterrand University Hospital, 21079 Dijon, France

**Keywords:** lumbar radiculopathy, MRI, coronal STIR, imaging, low back pain

## Abstract

Objective. Lumbar radiculopathy mainly originates in the spine (lumbar disc herniation or spine osteoarthritis) but can sometimes be explained by extra-spinal nerve compression or confused with referred pain mimicking radiculopathy. Our main objective was to demonstrate the clinical benefit of the large-field coronal STIR (coroSTIR) sequence in the etiological assessment of lumbar radiculopathy with a duration of more than six weeks. Materials and methods. Six hundred consecutive lumbar MRI scans performed using the same protocol were retrospectively reviewed. Two musculoskeletal radiologists independently assessed the coroSTIR sequence for the presence of extra-spinal anomalies (ESA) that could explain or contribute to the lumbar radiculopathy. The presence of an ESA was then correlated with sex, age, topography and lateralization of radiculopathy, history of vertebral surgery, as well as the presence of a spinal cause explaining the symptoms. Extra-spinal incidentalomas (ESI) with potential clinical impact visible only on the coroSTIR sequence were also systematically reported. Results. An extra-spinal cause was detected on the coroSTIR sequence in 68 cases (11.3%), mainly gluteal tendinobursitis (30.9%), congestive hip osteoarthritis (25%), degenerative sacroiliac arthropathy (14.7%), or inflammatory sacroilitis (7.3%). Their prevalence was significantly correlated in multivariate regression with age (58 years vs. 53 years, *p* = 0.01), but not with the type of radiating pain (sciatica or cruralgia). The presence of ESI was also frequent (70 cases, 11.7%), including some potentially severe diagnoses (38% of tumor or pseudo-tumor mass requiring further assessment or monitoring). Conclusions. Considering its acceptable acquisition time, the detection of a significant number of potentially symptom-related extra-spinal anomalies, and the discovery of a non-negligible number of extra-spinal incidentalomas with potential clinical impact, the coronal STIR should be performed systematically in routine MRI for lumbar radiculopathy.

## 1. Introduction

Lumbar pain with radiation in the lower limbs (buttock, thigh and/or leg) is a common complaint in medical consultations in primary care. It has been shown that radiculopathy in the lower limbs has an impact on prognosis and the overconsumption of health care compared to common low back pain [1,2]. The prevalence of radiculopathy is estimated to be between 4 and 6% in the adult population, and the individual risk of developing it during life is about 40% [3]. 

Currently, in the presence of red flags (altered general condition, traumatic event, fever, etc.) or when disabling pain persists despite appropriate medical treatment for 6 weeks, MRI is the imaging investigation of choice for establishing the correct diagnosis [4,5,6]. When searching for a concordant etiology, the assessment usually includes sagittal sequences in T1, T2 weighting without and with the saturation of the fat signal, and axial T2-weighted sequences on the different lumbar regions to accurately depict disco-radicular impingement [7]. 

However, in the large majority of lumbar radiculopathy results from disco-radicular anomalies at a lumbar spine level (for example, a herniated disc, lumbar spinal stenosis, or lumbar facet syndrome), true sciatica or cruralgia may result from the involvement of the roots in an extraspinal location. In addition to extraspinal etiology [8], some painful conditions located in the pelvic girdle—including bone fractures, sacroiliac, pubic or coxofemoral joint diseases, and gluteal or proximal hamstring tendinopathy—may be confused with radiculopathy [9,10,11] because they mimic the clinical symptoms of radiating pain toward the lower limbs, especially the buttock or thigh.

Nevertheless, routine lumbar MRI protocols explore only the lumbar spine and do not provide any information on the lumbosacral plexus or neighboring pelvic girdle. Thus, exploring these conditions requires at least an MRI sequence centered on the abdomen and pelvis, regions that are incompletely explored with the usual protocol. The coronal STIR sequence [12] has demonstrated its value for the study of these regions (sacroiliac, hip, trochanteric pain), notably through its ability to detect edema/inflammation [13,14].

To our knowledge, only one study has investigated the value of the coronal STIR sequence in a review of low back pain with radiculopathy: Laporte et al. suggested that this sequence should be performed essentially when no obvious radicular conflict had been demonstrated on the usual sequences centered on the lumbar spine and lumbosacral hinge [15]. As a continuation of this study, it seems interesting to us to evaluate the systematic use of this MRI sequence on a larger number of unselected patients (sciatica or cruralgia) to clarify its diagnostic relevance.

The main objective of our study was thus to demonstrate the clinical benefit of the large-field coronal STIR sequence in the etiological assessment of lumbar radiculopathy with a duration of more than 6 weeks. The secondary objective was to search for possible correlations between the discovery of extra-spinal anomalies (ESA) on this coroSTIR sequence with clinical elements or anamnestic elements (such as the topography of the radiation), as well as to identify the number of extra-spinal incidentalomas (ESI) with potential impact on further management.

## 2. Materials and Methods

This was a monocentric, non-interventional, observational study in routine care with a retrospective review of MRI data. All the patients signed a free informed written consent form explaining that their examination could be processed or reviewed for research purposes (with data anonymization) after the MRI had been performed.

### 2.1. Study Population

From May to October 2022, patients referred to the radiology department of the Valmy clinic on the Dijon-Bourgogne private hospital site for lumbar spine MRI following a complaint of low back pain with radiculopathy (>18 years) were included.

The exclusion criteria were the presence of clinical red flags justifying MRI (altered general condition, fever, trauma, etc.), the absence of a coronal STIR sequence in the MRI protocol, and the presence of artifacts making MRI interpretation difficult.

### 2.2. MRI Protocol

All examinations were performed on one of the two 1.5 Tesla MRIs, Voyager G3 model, from General Electric (GE). Two types of protocols have been used with a common feature being the systematic presence of a large FOV STIR coronal sequence centered on the abdominal cavity and pelvis (including the sacroiliac joints, coxo-femoral joints, and peri-trochanteric regions), as well as a sagittal T1 sequence centered on the lumbosacral spine. The protocols also included either a 3D T2 sequence (for 272 patients, 45.3%), or a sagittal T2 DIXON or STIR sequence on the lumbar spine with axial reconstructions on the last four lumbar stages (for 328 patients, 54.7%). The characteristics of the different MRI sequences are reported in Table 1.

### 2.3. Data Processing

Data concerning sex, age, weight, the topography of pain radiation (sciatica or cruralgia), the lateralization of pain, and the surgical history on the lumbar spine were collected.

Two radiologists, experts in musculoskeletal imaging with, respectively, 5 and 7 years’ experience in musculoskeletal imaging, independently analyzed the MRI images. Each radiologist performed two types of reading on each examination:

– The first reading involved the usual sequences centered on the lumbar spine, indicating whether or not there was an intervertebral disc hernia and/or spinal osteoarthritis that could explain the clinical lumbar radiculopathy.
– The second reading involved only the coronal STIR sequence, indicating whether or not there was an ESA that could explain the clinical complaint and whether there was any clinically significant ESI unrelated to the reported symptoms.

A third expert radiologist with more than fifteen years’ experience in musculoskeletal imaging was called in to resolve any diagnostic discrepancies between the first two readers.

The primary endpoint was considered positive when the CoroSTIR sequence found a significant ESA, whereas the standard protocol did not detect any spinal abnormality that could be responsible for or in association with the reported symptoms. 

### 2.4. Statistical Analysis

Statistical analyses were performed using the software (version 16) StataCorp LLC (College Station, TX, USA).

After an initial meeting between the two radiologists to confirm the reading methodology, the inter-observer reproducibility at the end of the study was excellent, with an overall kappa coefficient of 0.89 for the analyzed exams. The third expert reader intervened in 14 difficult cases to decide on the final diagnosis.

Categorical variables were presented using numbers and percentages, and quantitative variables were presented using numbers and interquartile ranges (IQR).

The Chi^2^ and Fisher’s exact tests, as well as an ROC curve, were applied to correlate the presence of an ESA with various collected data, such as age, sex, weight, the lateralization and location of the pain, the presence of a spinal lesion likely to explain the symptoms, and the history of lumbar surgery. An additional multivariate analysis was carried out using a logistic regression method, taking into account variables that were significant (or approaching significance) in univariate analysis.

## 3. Results

Six hundred patients (57% women; mean age of 54 years) were included in this real-life observational study. The baseline characteristics are detailed in Table 2. Among the 600 patients evaluated, 68 patients (11.33%, 95%CI [8.90–14.15%]) were found to have an anomaly visible on the coronal STIR sequence, potentially explaining the symptoms. This anomaly was associated with a spinal cause for 36 patients on the usual sequences (52.9%) or without any spinal cause for 32 patients (47.1%).

In univariate analysis, the presence of a coroSTIR anomaly was significantly associated with older age (mean age, 58 years vs. 53 years, *p* = 0.03) and with the absence of relevant findings in the MRI of the lumbar region (spinal MRI abnormality, 52.9 vs. 71.4%). An anomaly was found more frequently in the case of cruralgia than sciatica (23.5% vs. 14.5%), although the result was only approaching the significance threshold (*p* = 0.07).

In multivariate linear regression, age was the only factor that remained significantly associated with the presence of a coroSTIR anomaly (*p* = 0.01, area under the curve of 0.67).

**Figure 1:**
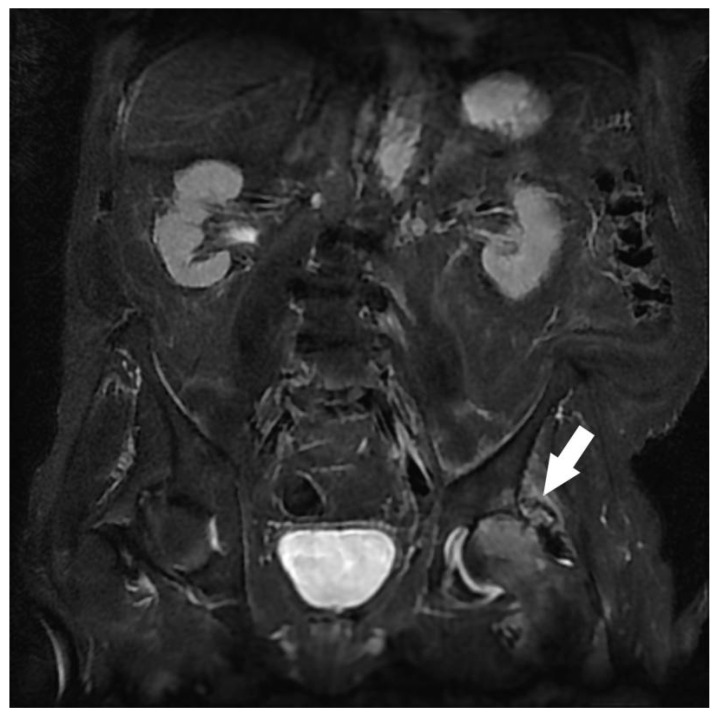
Assessment of left sciatica in an 87-year-old man. Coronal STIR MRI. Left coxo-femoral hypersignal predominant on the femoral head, narrowing of the joint space mainly supero-external, significant joint effusion, and degenerative hypertrophy of the labrum. Congestive left hip osteoarthritis (white arrow).

**Figure 2:**
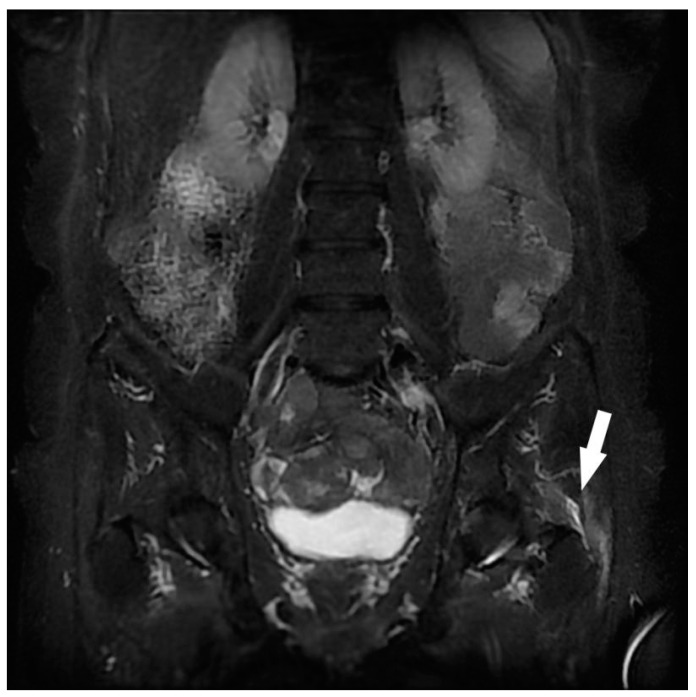
Assessment of left sciatica in a 72-year-old woman. Coronal STIR MRI. Hypersignal at the height of the left peri-trochanteric region, of the small and medium gluteal tendons with effusion within their purse. Left gluteal tendinobursitis (white arrow).

**Figure 3:**
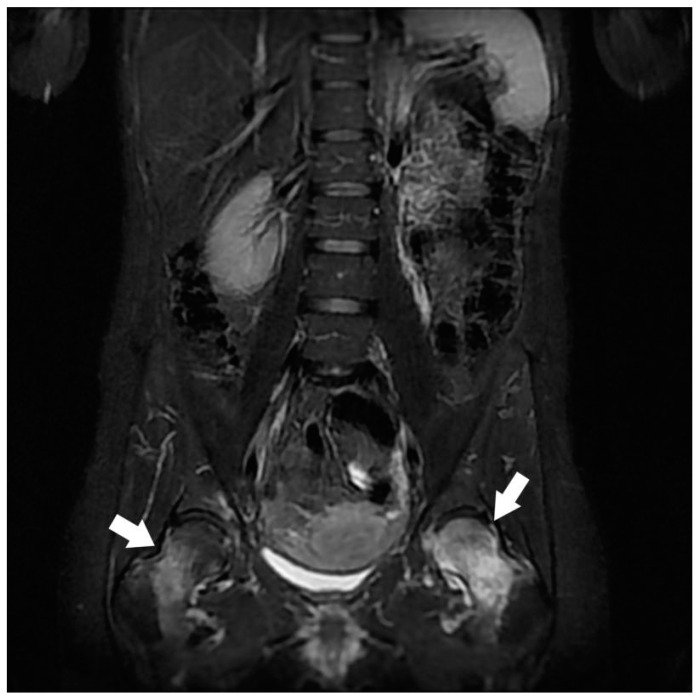
Assessment of bilateral sciatica in a 40-year-old woman in a postpartum context. Coronal STIR MRI. Bilateral hypersignal essentially involving the proximal ends of the femurs (white arrows) and, to a lesser extent, the bottom of the acetabulum. Predominant involvement on the left with joint effusion. Respect of the coxo-femoral joint spaces. Transient migrant regional osteoporosis.

**Figure 4:**
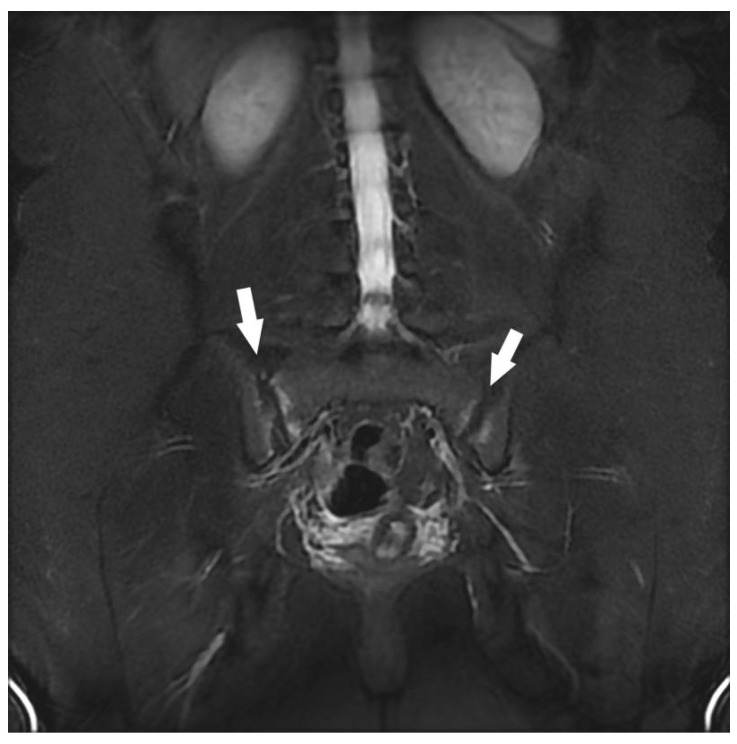
Assessment of right sciatica in a 27-year-old woman. Coronal STIR MRI. Bilateral hypersignal of subchondral bone (white arrows) involving the sacro-iliac joints (predominant on the right) with bone erosions of the right iliac edge.

**Figure 5:**
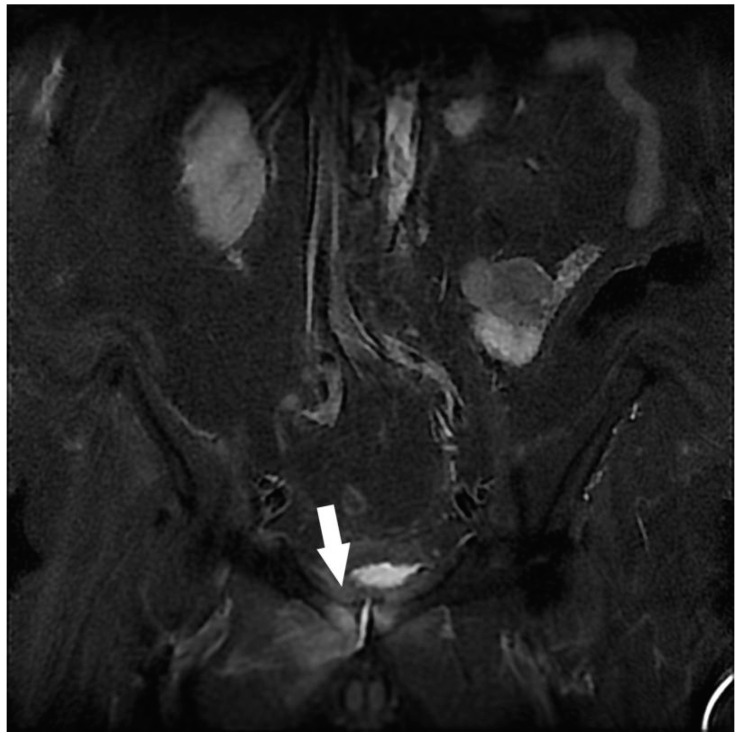
Assessment of right sciatica in an 80-year-old man two months after prostatectomy. Coronal STIR MRI. Hypersignal in mirror with effusion of the pubic symphysis. Inflammatory hypersignal in the proximal part of the adductor muscles, predominant on the right (white arrow). Septic pubic arthritis (confirmed by subsequent biopsy).

**Figure 6:**
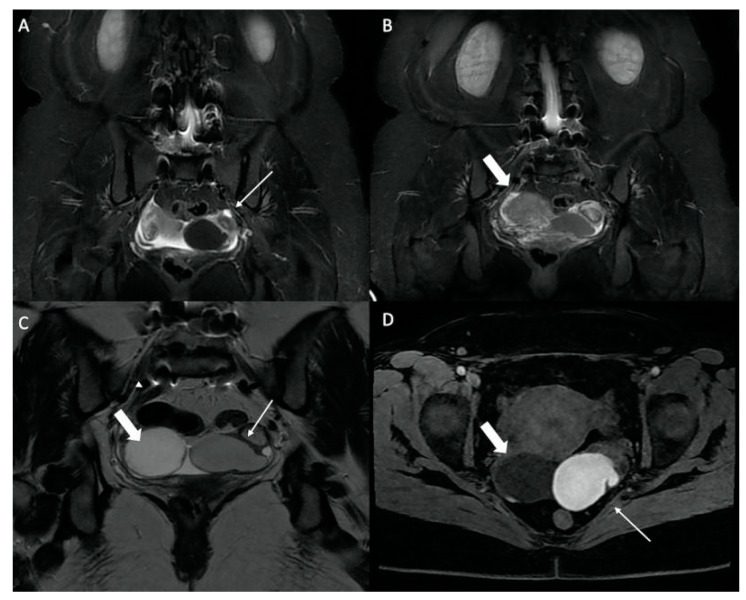
Assessment of bilateral sciatica in a 44-year-old woman. (**A**) Coronal STIR MRI. Left ovarian lesion in homogeneous STIR hyposignal (small arrow) with low abundance pelvic fluid effusion. (**B**) Coronal STIR MRI. Right adnexal lesion with heterogeneous signal (thick arrow). (**C**) T2 coronal MRI and (**D**) T1 axial MRI with saturation of the fat signal (complementary remote assessment). Right ovarian cystic lesion in T1 hyposignal, homogeneous T2 hypersignal, with small dependent hemorrhagic contingent in T1 hypersignal (thick arrows). Left ovarian lesion with “dirty” intermediate T2 signal with T2 shading sign, in spontaneous T1 hypersignal (small arrows). Aspect of “kissing ovaries” with ovarian adhesion in retro-uterine position. Left endometrioma and posterior deep pelvic endometriosis. Hemorrhagic right ovarian cyst.

All of the different etiologies (ESA) are presented in Table 3, and several examples are shown in Figure 1, Figure 2, Figure 3, Figure 4, Figure 5 and Figure 6 for illustrative purposes. The most frequent conditions mimicking lumbar radiculopathy pain were isolated gluteal tendinobursitis (n = 20, 30.9%), congestive hip osteoarthritis (n = 17, 25%), or congestive degenerative sacroiliac arthropathy (n = 10, 14.7%). It should be noted that five cases of inflammatory sacroiliitis (including one associated with gluteal tendinobursitis) were diagnosed, which are relevant differential diagnoses with specific potential targeted treatments. Rare abdominopelvic lesions were also discovered, such as a large endometrioma or a retroperitoneal mass syndrome with unilateral urinary obstruction.

An ESI with potential clinical impact (details in Table 4) was discovered on the coroSTIR sequence in 70 patients (11.7%, 95%CI [9.2–14.5%]), including some potentially severe diagnoses (38% tumor or pseudo-tumoral mass requiring further assessment or monitoring). Two-thirds concerned the pelvic region, predominantly gynecological or prostatic abnormalities. 

## 4. Discussion

The value of additional MRI sequences, such as coronal STIR in the investigation of lumbar radiculopathy, has been scarcely reported in the literature so far (15). This study illustrates that there may be overlap in the lumbar radiculopathy between discoradicular impingement and other causes of radiating pain in an extraspinal location, including troncular nerve entrapment or differential diagnoses in a large cohort of 600 patients consecutively included. Indeed, our results demonstrate for the first time the clinical benefit of the large-field coronal STIR sequence in the etiological assessment of lumbar radiculopathy in real-life practice. This was achieved by including all extra-spinal anomalies that could explain the clinical complaints, regardless of whether the lumbar sequences showed a disc hernia and/or spinal osteoarthritis that can already explain the clinical lumbar radiculopathy. 

The systematic use of the coronal STIR sequence in the MRI assessment of lumbar radiculopathy in 600 patients led to the discovery of ESA in 11.3% (68/600) of cases that could fully or partially explain the clinical symptomatology. This incidence is higher than those measured in the few studies in the literature dealing with the subject. Gleeson et al. [16] found 2.7% (7/260) of ESA on the coronal STIR sequence in a work-up of low back pain with or without radiculopathy, but this study focused only on the sacroiliac joints, which is consistent with our results (15/600 or 2.5% sacroiliac abnormalities). In a review of low back pain patients with or without radicular pain, Gupta et al. [17] detected that 6.8% (24/350) of ESA was potentially related to the clinical symptoms. However, no abnormality in the peri-trochanteric regions was mentioned, which seems unlikely given the very high frequency of this condition after 50 years, isolated or associated with lumbar radiculopathy (the gluteal muscles being innervated via the L4, L5, and S1 roots). In our cohort, 20 cases of trochanteric tendinobursitis were noted, which can frequently mimic lumbar radiculopathy for the clinician whose management is different from lumbar radiculopathy originating in the spine.

The study by Laporte et al. [15], whose inclusion criteria were similar to ours, reported 5.7% (12/209) of clinically related ESA. Unlike Laporte et al., we made the choice to include ESA that seemed significant, even if there was already a spinal cause that could explain the symptoms of the radiculopathy. This was the case in our study for 36 patients (52.9% of ESA cases), which might explain the differences with the results reported by Laporte et al. (16.7% of cases).

Moe [recently, Romeo et al. [18]] studied the coroSTIR sequence in the assessment of isolated low back pain (without radiculopathy) with less convincing results, detecting ESA in only 3.5% (35/931) of cases. This underlines the relevance of performing coroSTIR when low back pain is associated with radiculopathy, probably even more so for cruralgia pain than for lumbosciatica pain because differential diagnoses are more frequent in routine care (hip conditions sometimes resulting in clinical pictures of pseudo-cruralgia). The lack of knowledge of the exact radiation territory in many patients when the clinician prescribes MRI reduces the statistical power on this secondary endpoint, which therefore needs to be confirmed in other cohorts.

As reported by Laporte et al. [15], age is significantly correlated in multivariate analysis with the presence of ESA on this sequence, because the prevalence of degenerative musculoskeletal conditions increases sharply after 60 years, which justifies extra vigilance from the radiologist, especially since the risk of ESI also increases (especially for tumors).

The clinically significant ESI rate of 11.7% in our study is consistent with the only two studies in the literature that have specifically addressed this issue, despite the arbitrary nature of the potential clinical impact and its precise definition. In Seeman et al., ESI prevalence was 22%, including 7.8% with potential clinical impact (236/3024) [19]. Khasawneh et al. found a total of 31.1% of ESI, including 8.7% with potential clinical impact (131/1509) [20]. Similar to our results, these two studies underline a significant lack of reporting of these ESI in radiologist reports, even though they can lead to additional investigations and potentially serious diagnoses (38% of mass tumor or pseudo-tumor here). This underlines the importance of a systematic, region-by-region, standardized analysis of coronal STIR sequences.

Several limitations of the present study are inherent to its monocentric and retrospective nature, including the absence of patient follow-up. We were therefore unable to establish a correlation between the diagnoses suggested by ESA and the clinical symptoms or to assess longer-term patient outcomes after appropriate treatment. This could lead to the overdiagnosis of ESA, particularly due to the high sensitivity of gluteal tendinobursitis on MRI, sometimes without clinical relevance. Indeed, in elderly populations, the presence of peritrochanteric T2 signal abnormalities are found in up to 50% of asymptomatic people [21]. This is illustrated in our study by the number of ESI (n = 10) for contralateral gluteal tendinobursitis, which does not explain the symptoms that led to the MRI assessment. Another limitation could be the acquisition time of an additional CoroSTIR sequence, but the feasibility remains good as it does not significantly extend the image acquisition time (<3 min).

Another limitation due to a lack of sensitivity is the possibility of an underdiagnosis of small truncal nerve lesions (such as schwannoma or neuroma) or of a piriformis syndrome due to the limited spatial resolution of this MRI sequence. With the recent progress of new diffusion tensor MRI sequences of nerve tractography [22,23], in the future we should be able to detect these rare lesions in complex cases of radiating lower limb pain associated with normal spinal MRIs.

Our results raise the question of systematically adding a coroSTIR sequence in the assessment of lumbar radiculopathy. Although this sequence increases the duration of the examination by approximately two minutes, our results suggest that it is justified and relevant. More recently, Zanchi and Solmann have highlighted the benefits of using a simplified protocol with a sagittal T2 Dixon sequence to replace the standard protocol (sagittal T1, T2 sequences without and with fat signal suppression) in order to reduce examination time and improve patient comfort [24,25]. The evaluation of this sagittal T2 Dixon sequence in association with the coronal STIR large FOV sequence could be interesting in the future for the most exhaustive exploration possible in the shortest time, which remains to be confirmed.

In conclusion, systematically performing the coronal STIR sequence centered on the abdomino-pelvic cavity and pelvis for the assessment of lumbar radiculopathy can detect a significant number of extra-spinal anomalies that may be related to the symptoms. Moreover, there was a fortuitous discovery of extra-spinal incidentalomas with potential clinical impact in a non-negligible number of cases. These results reinforce the diagnostic value of coronal STIR in the etiological assessment of lumbar radiculopathy, and should be performed systematically in routine lumbar MRI.

## Figures and Tables

**Table 1:** MRI data. Voyager 1.5T GE Healthcare Deep Learning (×2).

MRI Sequences	TE (ms)	RT (ms)	FOV (cm)	Gap (mm)	Thickness (mm)	Matrix	Acquisition Time (min)
3D T2	137	1000	34	0.5	1	340 × 340	03:36
Sag T1	8	453	35	0.5	3.5	416 × 320	00:55
SagT2 DIXON	85	3014	25	0.4	3.5	288 × 232	02:47
Sag STIR	104	2994	35	0.5	3.5	340 × 320	02:33
Coro STIR	130	6987	40	1	6.5	292 × 292	01:45
Ax T2	108	2531	18	1	4	288 × 256	02:12

**Table 2:** Characteristics of patients according to the presence of an anomaly in large-field CoroSTIR sequence.

	Total (n = 600)	CoroSTIR – (n = 532)	CoroSTIR + (n = 68)	*p*-Value
Patients				
Gender (%M/F)	42/57	43/57	40/60	0.69
Age	54.1 [IQR 43-66]	53.5 [IQR 42-65]	58.8 [IQR 46-66]	0.01
Weight	76.9 [IQR 67-85]	76.8 [IQR 67.5-85]	77.7 [IQR 66-86.5]	0.57
ATCD lumbar surgery (%)	11.2	11.7	7.4	0.41
Clinical				
Lateralization	D: 219 (36.5%)	D: 194 (36.5%)	D: 25 (36.8%)	0.81
	G: 254 (42.3%)	G: 227 (42.7%)	G: 27 (39.7%)	
	B: 127 (21.2%)	B: 111 (20.9%)	B: 16 (23.5%)	
Root topography				
Cruralgia	93 (15.5%)	77 (14.5%)	16 (23.5%)	0.07
Sciatica	213 (35.5%)	193 (36.3%)	20 (29.4%)	0.28
Buttock pain or truncated sciatica	248 (41.3%)	221 (41.5%)	27 (39.7%)	0.79
Radiating pain without precision	3 (0.5%)	2 (0.4%)	1 (1.5%)	0.30
Not known	49 (8.2%)	44 (8.3%)	5 (7.4%)	1
Imaging				
Lumbar MRI cause to symptoms	416 (69.3%)	380 (71.4%)	36 (52.9%)	0.003

D: right; L: left; B: bilateral.

**Table 3:** Summary of ESA from the large-field coroSTIR sequence explaining (11.7% of MRIs analyzed).

Hip Joint	Congestive hip osteoarthritis	**17**
	Stress femoral fracture	4
	Avascular femoral osteonecrosis	1
	Extensive acetabular edema	1
Gluteal Muscles	Gluteal tendinobursitis	21
Sacroiliac Joint	Congestive degenerative arthropathy	10
	Inflammatory sacroiliitis	4
	Sacral fracture	1
Pubis	Septic arthritis of the pubis	1
Femur	Periprosthetic loosening fracture (total hip replacement)	1
	Greater trochanter fracture	1
Mixed Musculoskeletal Causes	Inflammatory sacroiliitis and gluteal tendinobursitis	1
	Degenerative congestive sacroiliac arthropathy and gluteal tendinobursitis	1
Others	Endometrioma with posterior deep pelvic endometriosis	1
	Ischio-femoral impingement	1
	Retroperitoneal mass syndrome with urinary obstruction	1

**Table 4:** ESI with potential clinical impact (n = 70, 11.7% of MRIs analyzed).

Kidney	Atypical renal lesion ^1^	**9**
	Multicystic dysplasia, Polycystic kidney disease	5
Liver	Non-cystic liver lesion ^1^	2
Pelvis	Cystic or non-cystic ovarian lesion ^1^	15
	Prostatomegaly with struggle bladder	11
	Polymyomatous uterus	4
	Atypical uterine myoma ^1^	2
	Indeterminate pelvic mass ^1^	1
	Diffuse uterine adenomyosis	1
Musculotendinous	Gluteal tendinobursitis ^2^	10
	Denervation edema on chronic root conflict ^2^	1
	Intramuscular lesion (gluteus maximus) ^1^	1
	Proximal iliotibial band enthesopathy ^2^	1
Osteoarticular	Congestive hip osteoarthritis ^2^	8
	Congestive degenerative sacroiliac arthropathy ^2^	8
	Atypical bone lesion (iliac wing, femoral neck, sacrum) ^1^	3
	Severe hip dysplasia ^2^	1
	Congestive pubic osteoarthritis ^2^	1
	Avascular femoral osteonecrosis ^2^	1
Others	Splenomegaly	1

^1^ requiring additional assessment or monitoring. ^2^ anomaly contralateral to symptoms.

## Data Availability

Not applicable.
